# DJ-1 administration exerts cardioprotection in a mouse model of acute myocardial infarction

**DOI:** 10.3389/fphar.2022.1002755

**Published:** 2022-09-23

**Authors:** Alex Gallinat, Guiomar Mendieta, Gemma Vilahur, Teresa Padró, Lina Badimon

**Affiliations:** ^1^ Cardiovascular Program-ICCC, IR-Hospital Santa Creu i Sant Pau, IIB-Sant Pau, Barcelona, Spain; ^2^ Universitat Autònoma de Barcelona (UAB), Barcelona, Spain; ^3^ Centro Nacional de Investigaciones Cardiovasculares Carlos III (CNIC), Madrid, Spain; ^4^ CIBERCV-Instituto de Salud Carlos III, Madrid, Spain; ^5^ Cardiovascular Research Chair, UAB, Barcelona, Spain

**Keywords:** cardioprotection, DJ-1, PARK7, ischemia, reperfusion, myocardial infarction, ischemia/reperfusion injury

## Abstract

Cardiovascular diseases, and particularly acute myocardial infarction (MI), are the most common causes of death worldwide. Infarct size is the major predictor of clinical outcomes in MI. The Parkinson’s disease associated protein, DJ-1 (also known as PARK7), is a multifunctional protein with chaperone, redox sensing and mitochondrial homeostasis activities. Previously, we provided the evidence for a central role of endogenous DJ-1 in the cardioprotection of post-conditioning. In the present study, we tested the hypothesis that systemic administration of recombinant DJ-1 exerts cardioprotective effects in a mouse model of MI and also explored the associated transcriptional response. We report a significant treatment-induced reduction in infarct size, leukocyte infiltration, apoptosis and oxidative stress. Effects potentially mediated by G-protein-coupled receptor signaling and modulation of the immune response. Collectively, our results indicate a protective role for the exogenously administrated DJ-1 upon MI, and provide the first line of evidence for an extracellular activity of DJ-1 regulating cardiac injury *in vivo*.

## Introduction

Cardiovascular diseases are the leading cause of mortality worldwide. Among them, ischemic heart disease is the most common pathology (Benjamin et al., 2017). Myocardial infarction (MI) is defined as ischemia of the myocardial tissue, commonly caused by the occlusion of the coronary artery. Consequently, necrosis develops within the myocardium at risk (Reimer et al., 1979). Therefore, both the duration and the severity of the ischemic insult are major determinants of the final infarct size. Due to the limited regenerative capacity of the adult human heart, there is little replacement of lost functional tissue following MI. Rather, nearby fibroblasts are activated to replace the damaged myocardial tissue with fibrous tissue (Frangogiannis, 2006; Aix et al., 2018). Since the scar lacks contractile function, it decreases cardiac output, eventually leading to heart failure and free wall rupture (Kutty et al., 2013), Therefore, infarct size is the major predictor of clinical outcomes following MI (Sobel et al., 1972; Kelle et al., 2009).

Considering the time-dependent transition from ischemia to necrosis of the myocardium at risk, early reperfusion remains the definitive treatment for the ischemic heart. However, the rapid transition from ischemia to normoxia during reperfusion carries the potential to exacerbate damage in a process known as ischemia/reperfusion (I/R) injury (Yellon and Hausenloy, 2007). This is in part related to the generation of reactive oxygen species (ROS) (Zweier, 1988) affecting, among others, the integrity of the sarcoplasmic reticulum which leads to calcium overload, hyper-contracture, and the opening of the mitochondrial permeability transition pore, eventually causing cell death. Also, the changes in intracellular pH and the triggered immune response compromise the viability of the myocardium upon reperfusion (Lemasters et al., 1996; Liu et al., 2016). The aforementioned detrimental effects of reperfusion occur within minutes of restoration of coronary flow and account for a significant part of the final infarct size (Yellon and Hausenloy, 2007; Garcia-Dorado et al., 2009). A large number of pharmacological agents have shown to reduce infarct size in pre-clinical studies, but they have failed to demonstrate significant clinical benefits (Hausenloy et al., 2013; Heusch, 2017). Thus, cardioprotection is currently an unmet clinical need.

The early-onset Parkinson’s disease associated protein DJ-1 (also known as PARK7) is a multifunctional protein with cardioprotective effects against I/R injury (Dongworth et al., 2014; Shimizu et al., 2016; Shimizu et al., 2020) and oxidative stress (Billia et al., 2013). In a previous study, where the mitochondrial proteomic response to I/R and post-conditioning was analysed in a pre-clinical model of MI, we provided evidences for a central role of DJ-1 in the cardioprotection conferred by post-conditioning (Gallinat et al., 2022). The reported functions of DJ-1 include chaperone (Shendelman et al., 2004), protease (Chen et al., 2010), and deglycase (Richarme et al., 2015; Richarme and Dairou, 2017) activities, regulation of transcription (Takahashi-Niki et al., 2017), redox sensing (Wilson, 2011) and modulation of mitochondrial homeostasis (Hayashi et al., 2009; Heo et al., 2012). Also, some reports suggest extracellular activities for DJ-1. It is secreted under several pathological conditions including breast cancer (Le Naour et al., 2001), Parkinson’s disease (Maita et al., 2008; Tsuboi et al., 2008) or stroke (Allard et al., 2005), and a protective role against ischemia (Kaneko et al., 2014) and I/R (Han et al., 2017). Likewise, we have reported endothelial DJ-1 secretion under ischemia and I/R, and evidenced its effects on endothelial cell function at reperfusion, thereby suggesting a role in regulating cardiac injury (Gallinat and Badimon, 2022). In this study, we tested the hypothesis that systemic administration of recombinant protein DJ-1 exerts a cardioprotective effect in a mouse model of acute MI.

## Materials and methods

### Murine model of myocardial infarction

The present study was performed in male CH3 mice of 8–10 weeks’ old weighing 25–30 g (*n* = 26; Jackson Laboratory, Bar Harbor, ME, United States). Mice were randomly given an intraperitoneal injection of 50 μg of DJ-1 (*n* = 10; full length recombinant human DJ-1 > 95% purity, MBS143125, MyBioSource, San Diego, CA, United States); or equal PBS volume for vehicle/controls (*n* = 16), 60 min prior to the induction of MI by 45 min ligation of the left anterior descending coronary artery (LAD), as previously described (Cubedo et al., 2016; Mendieta et al., 2019). Briefly, animals were anesthetized with a mixture of O_2_/isoflurane, intubated and mechanically ventilated (rate 90 breath/min, tidal volume 0.1 ml; TOPO dual mode ventilator, Kent Scientific Corporation; Torrington, CT, United States). Core temperature was continuously monitored throughout the surgery and maintained within 37–38°C using a heat pad and heat lamp. An anterior thoracotomy was performed; the heart was exposed and the LAD coronary artery was occluded with an intramural stitch (7–0 silk suture) for 45 min. The success of complete coronary ligation was verified by electrocardiographic visualization of the ST-elevation-MI pattern that was continuously monitored and the visualization of a pale and hypokinetic ventricular region distal to the site of occlusion. After 45 min of ischemia, animals were sacrificed (Ischemia group; *n* = 11 vehicle and *n* = 5 DJ-1) or reperfused for 2 h (I/R group; *n* = 5 vehicle and *n* = 5 DJ-1). A sham-operated group (*n* = 4), which underwent the same surgical procedure without ligation of the LAD was included. Afterwards, hearts were carefully excised and processed for the following procedures. The dose of DJ-1 was chosen based on previous studies (Arac et al., 2011; Cubedo et al., 2016). All analyses were performed blindly with regard to the treatment received by the animals.

### Morphometric assessment of infarct size

Hearts (*n* = 26) were immersed in a fixative solution (4% paraformaldehyde), embedded in optimal cutting temperature compound (OCT) and sequentially cross-sectioned from the apex to the base (10 µm thick sections 200 µm distanced). Sections were then stained with haematoxylin-eosin and infarct size analysis was morphometrically determined with the image analysis software ImageJ (Schindelin et al., 2012). Infarct size was calculated as the sum of myocardial infarct areas between total left ventricle wall surface, as previously reported (Takagawa et al., 2007; Cubedo et al., 2016). Three measurements per each histological section were determined.

### Immunohistochemical analysis

OCT-embedded frozen specimens were cut into 5 μm thick serial sections, placed on poly-l-lysine coated slides, and processed for immunohistochemistry. The sections were incubated for 2 h with rabbit polyclonal antibodies against DJ-1 (AP01249PU-N; 1:50 dilution; Acris Antibodies GmbH, Herford, Germany), neutrophil elastase (ab68672; 1:100 dilution; Abcam, Cambridge, United Kingdom), inducible nitrogen oxide synthase (iNOS; NB300-605; 1:20 dilution; Novus Biologicals; Littelton, CO, United States) and cleaved caspase-3 (Asp175; 9,661; 1:200 dilution; Cell Signaling; Danvers; MA, United States), mouse monoclonal antibody against monocyte/macrophages (ab33451; 1:50 dilution; Abcam, Cambridge, United Kingdom), or goat polyclonal antibody against 8-hydroxy-deoxy-Guanine (8-OHdG; MBS536217; 1:300 dilution; MyBioSource, San Diego, CA, United States). Thereafter, sections were rinsed and incubated with the appropriate biotinylated antibodies (1:200 dilution; Vector Laboratories, Burlingame, CA, United States). Endogenous peroxidase activity, as well as unspecific unions were blocked before incubation with primary antibodies. Finally, sections were incubated with avidin-biotin complex (Vector Laboratories, Burlingame, CA, United States), and 3,3′-diaminobenzidine was used as the substrate for peroxidase. Images were acquired with a Nikon Eclipse 80i microscope (Nikon, Tokyo, Japan), digitized by a Retiga 1300i camera (Teledyne Photometrics, Tucson, AZ, United States), and imported to ImageJ (Schindelin et al., 2012). Positive signal was then quantified and expressed as the percentage of total area. For leukocyte infiltration and cleaved caspase-3 analyses, the number of positively labelled cells per field were counted. Six random fields per sample were analysed.

### Apoptosis assessment

Apoptosis was histologically analysed by the dUTP neck-end labeling (TUNEL) assay according to manufacturer’s specifications (Chemicon Inc.; Pittsburgh, PA, United States). The apoptosis rate was measured in a section below the occlusion and expressed as the percentage of TUNEL-positive cells per field (5 random fields per heart). All images were acquired in the same conditions.

### RNA extraction

Frozen tissue was grinded using mortar and pestle, and total RNA was extracted using a combined organic extraction and silica-membrane columns method (RNesasy Mini Kit, Qiagen; Valencia, CA, United States). RNA was then quantified by spectrophotometry using the Nanodrop ND-1000 (Thermo Fisher Scientific). RNA quality was assessed with the Agilent 2100 Bioanalyzer technology (Agilent Technologies; Santa Clara, CA, United States) and the Agilent RNA 6000 Nano Kit (Agilent Technologies; Santa Clara, CA, United States). Only RNA samples with an RNA Integrity Number (RIN) > 7 were chosen for microarray experiments.

### Transcriptomic and *in silico* analysis

Myocardial gene expression changes were analysed with a GeneChip Mouse 1.0ST array approach (Affymetrix, Santa Clara, CA, United States). Using the Ambion WT Expression Kit (Ambion, Life Tecnologies, Carlsbad, CA, United States) 100 ng of total RNA (mixed with poly-A controls; Affymetrix, Santa Clara, CA, United States) were retro-transcribed to double strand DNA, in two steps, in order to obtain cRNA. Single strand DNA was generated from 10 ug of cRNA. Then 5.5 µg of single strand DNA were fragmented and labelled with biotin using the WT Terminal Labeling Kit (Affymetrix, Santa Clara, CA, United States). Hybridization controls from the Hybridization, Wash and Stain Kit (Affymetrix, Santa Clara, CA, United States) were added to the sample. Thereafter, every sample was hybridized to a GeneChip Mouse Gene 1.0 ST array for 16 h at 45°C and 60 rpm, according to manufacturer’s instructions. Hybridization, washing, staining, and scanning of microarrays were performed according to Affymetrix instructions using the Affymetrix GeneChip 3000 7G System (645 Hybridization Oven, 450 Fluidic Station and GeneChip 3,000 7G Scanner). Raw data were pre-processed (background correction, normalization and median polish summarization of the probes) with Robust Multiarray Average (RMA) method. Microarray quality control and statistical analyses were performed using Expression Console (Affymetrix, Santa Clara, CA, United States) and the Partek Genomics Suite software (Partek Inc., St Louis, MI, United States). *p*-values were adjusted for multiple testing with Benjamini and Hochberg method, and false discovery rates (FDR) were calculated. Bioinformatic analysis was performed with WebGestAlt (ZhangLab; http://www.webgestalt.org/) following a Gene Set Enrichment Analysis (GSEA) (Xin et al., 2019) approach and defining gene ranks by log-fold change (logFC). Gene sets available from Wikipathways (Martens et al., 2021), Molecular Signature Data Base (MSigDB) (Liberzon et al., 2015), and Panther (Thomas et al., 2003) pathway collections were considered for the analysis. Raw expression data have been deposited in the NCBI’s Gene Expression Omnibus (GEO) (Edgar et al., 2002) and are accessible under the GEO Series accession number GSE66307.

### qPCR

Validation of myocardial gene expression changes was performed in tissue samples from the I/R group. *Gprc5a, Inos* and *Casp3* mRNA levels were analysed by real-time polymerase chain reaction, as previously described (Luquero et al., 2022). On-demand TaqMan RT-PCR assays for indicated genes were employed (Thermo Fisher Scientific Inc., Waltham, MA, United States).

### Statistical analysis

Because data were not normally distributed as assessed by the Shapiro-Wilk test, a non-parametric statistical analysis was employed. Non-parametric Kruskal-Wallis followed by Dunn’s test for multiple comparisons and Mann-Whitney test were used to assess differences between groups. Results are reported as median with inter-quartile range unless otherwise stated. Correlations were determined with Spearman’s rank correlation coefficient. All statistical analyses were performed with the statistical software package Statview 5.0.1 (SAS Institute Inc.; Cary, NC, United States).

## Results

### Systemic administration of recombinant DJ-1 protects the heart against I/R injury

**FIGURE 1:**
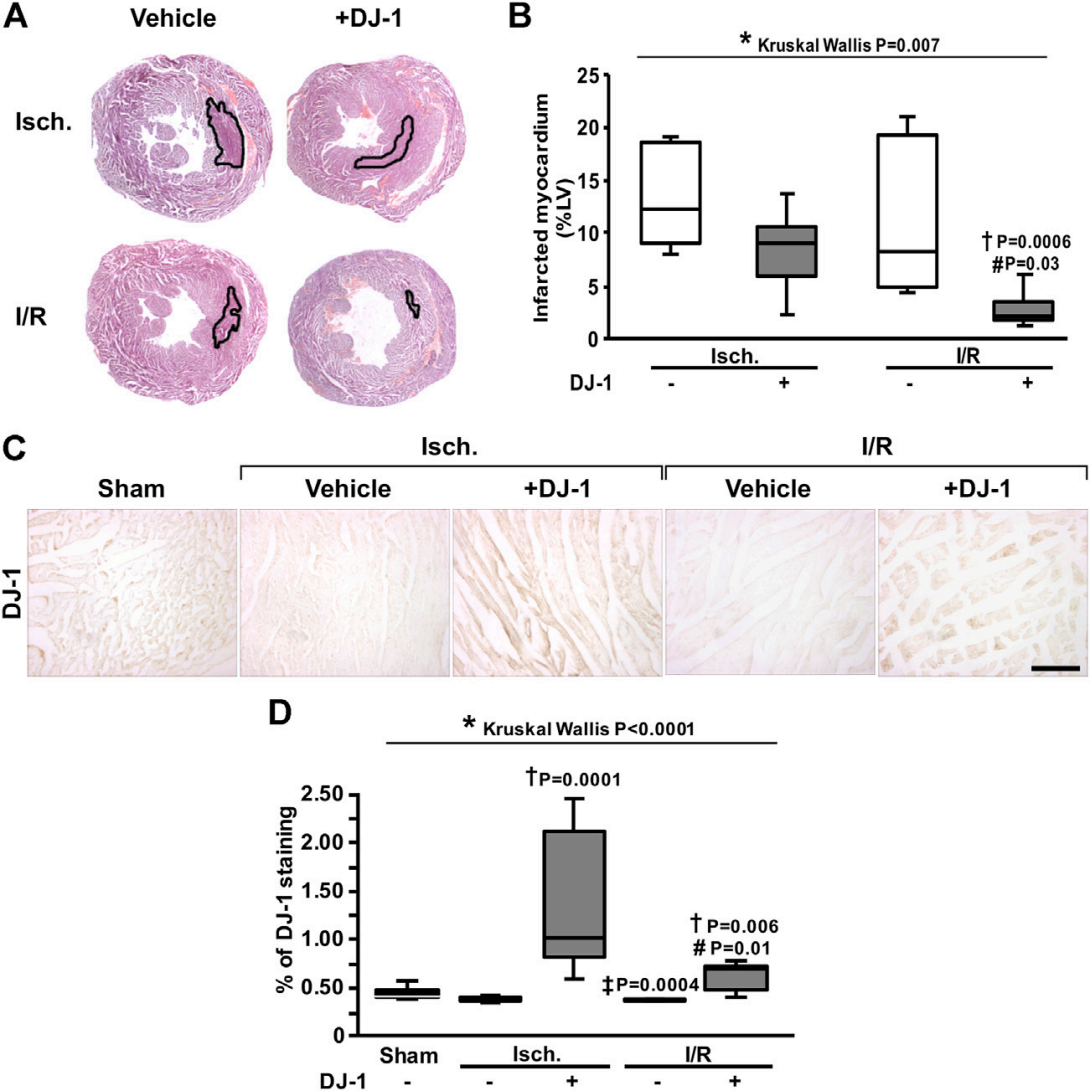
Effects of systemic DJ-1 administration on infarct size in a mouse model of MI. **(A)**. Representative images of infarcted heart sections. Infarct area is outlined in black. **(B)**. Infarct size quantification after ischemia and ischemia/reperfusion, in the presence and the absence of a systemic administration of DJ-1. Infarct measures are expressed as percentage of left ventricle area. **(C)**. Representative immunohistochemical acquisitions of DJ-1 in the myocardium after ischemia and ischemia/reperfusion, in the presence and the absence of a systemic administration of DJ-1. A sham-operated group was included as baseline. **(D)**. DJ-1 myocardium content quantification across groups. Scale bar: 100 µm; *p < 0.05, Kruskal-Wallis; †*p* < 0.05 vs. ischemia without DJ-1, ‡*p* < 0.05 vs. ischemia + DJ-1, #*p* < 0.05 vs. I/R without DJ-1, Dunn’s test. LV, Left Ventricle; Isch., Ischemia; I/R, Ischemia/Reperfusion.

In order to test whether the administration of DJ-1 exerts cardioprotection after MI, a group of mice were intraperitoneal treated with human recombinant DJ-1 (50 µg) 1 h before LAD coronary artery ligation, in a double-blind experimental design, and heart samples were processed for histological analysis. Computer-assisted morphometric assessment of infarct size revealed an infarct size reduction of about 75% for the animals treated with DJ-1 (Figure 1A,B). Interestingly, also a reduction in infarct size of about 20% was detected in the ischemia group, for the animals treated with DJ-1 (Figure 1A). Importantly, a 5.4-fold mean increase in the DJ-1 signal was found in the myocardium of the treated animals (Figure 1C,D), meaning the administrated DJ-1 reached the heart.

### DJ-1 administration modifies the myocardial transcriptomic response to I/R

**FIGURE 2:**
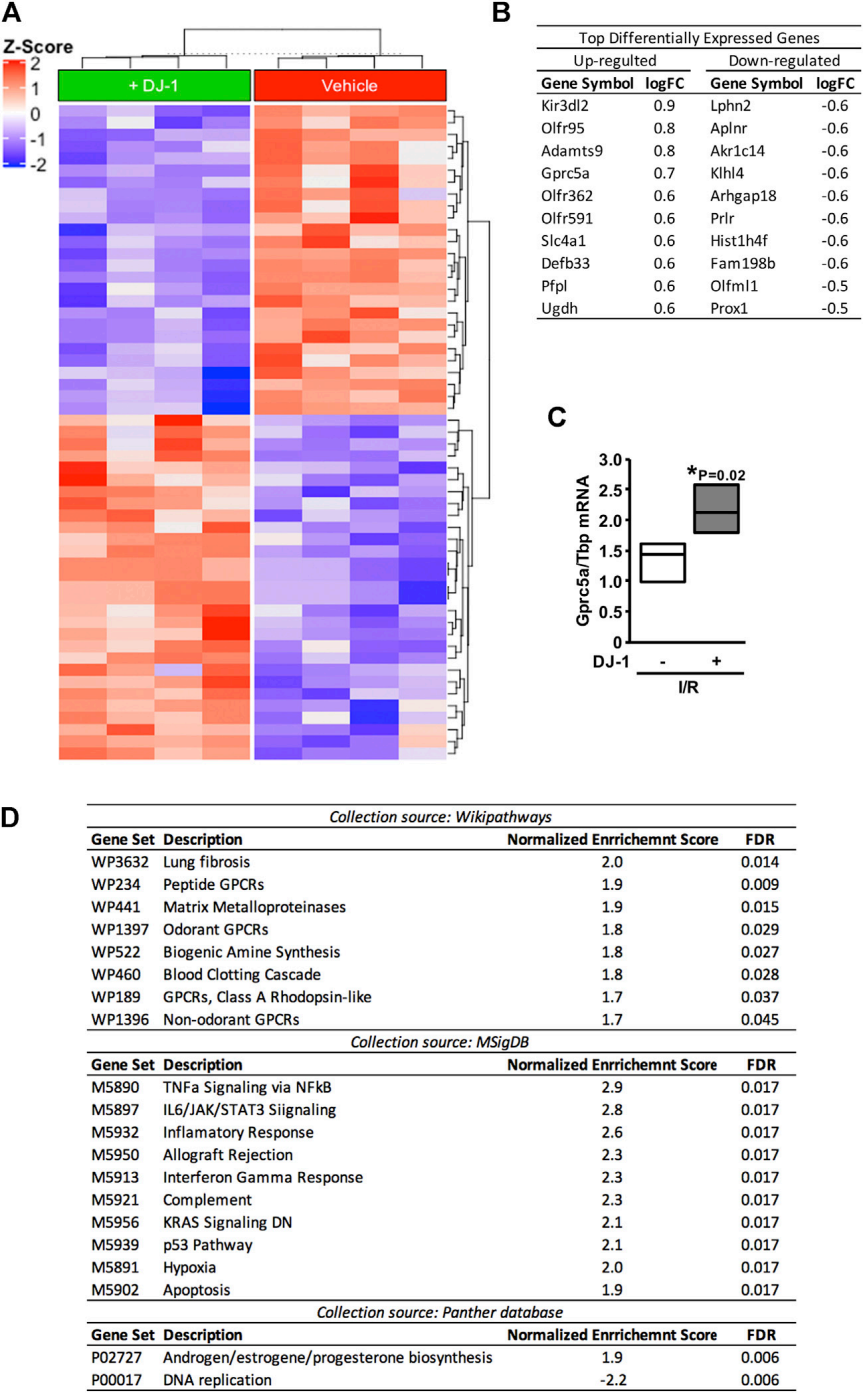
Differential gene expression profile after ischemia/reperfusion, in the presence and the absence of a systemic administration of DJ-1. **(A)**. Heat-map representation of differentially expressed genes. **(B)**. Top ten up- and down-regulated genes after ischemia/reperfusion in the presence of DJ-1 systemic administration. **(C)**. *Gprc5a* transcript analysis by qPCR. **(D)**. Gene-set enrichment analysis result. Gene-set collections from Wikipathways, Molecular Signature, and Panther databases were considered. *p < 0.05, Mann-Whitney. I/R, Ischemia/Reperfusion; logFC, log-Fold Change.

In order to outline possible mechanisms at play, we analysed the transcriptomic response of the myocardium upon MI in the presence and the absence of a systemic administration of DJ-1. As depicted in the heat-map, the administration of DJ-1 before I/R induced a multi-genic response in the myocardium different from that of the vehicle group (Figure 2A). Figure 2B shows the top 10 up- and down-regulated genes in the DJ-1-treated mice compared to the vehicle group.

Genome-wide RNA expression data was then analysed following a GSEA (Subramanian et al., 2005) approach considering all gene set collections available at Wikipathway (Martens et al., 2021), Molecular Signature (Liberzon et al., 2015), and Panther (Thomas et al., 2003) databases. As a result, different pathways were detected to be significantly enriched (FDR < 0.05) within each analysis (Figure 2D). From them all, G protein-coupled receptors (GPCR) mediated signaling and immune response-related gene sets were the most consistently detected. Interestingly, an apoptosis-related gene set was also significantly enriched (FDR = 0.017).

As a surrogate of the GPCR-mediated signaling-related gene set, the expression of *Gprc5a* gene was validated by qPCR (Figure 2C). Effects upon the immune response and apoptosis related gene sets were functionally validated as follows.

### Systemic DJ-1 administration reduces myocardial leukocyte infiltration following I/R

**FIGURE 3:**
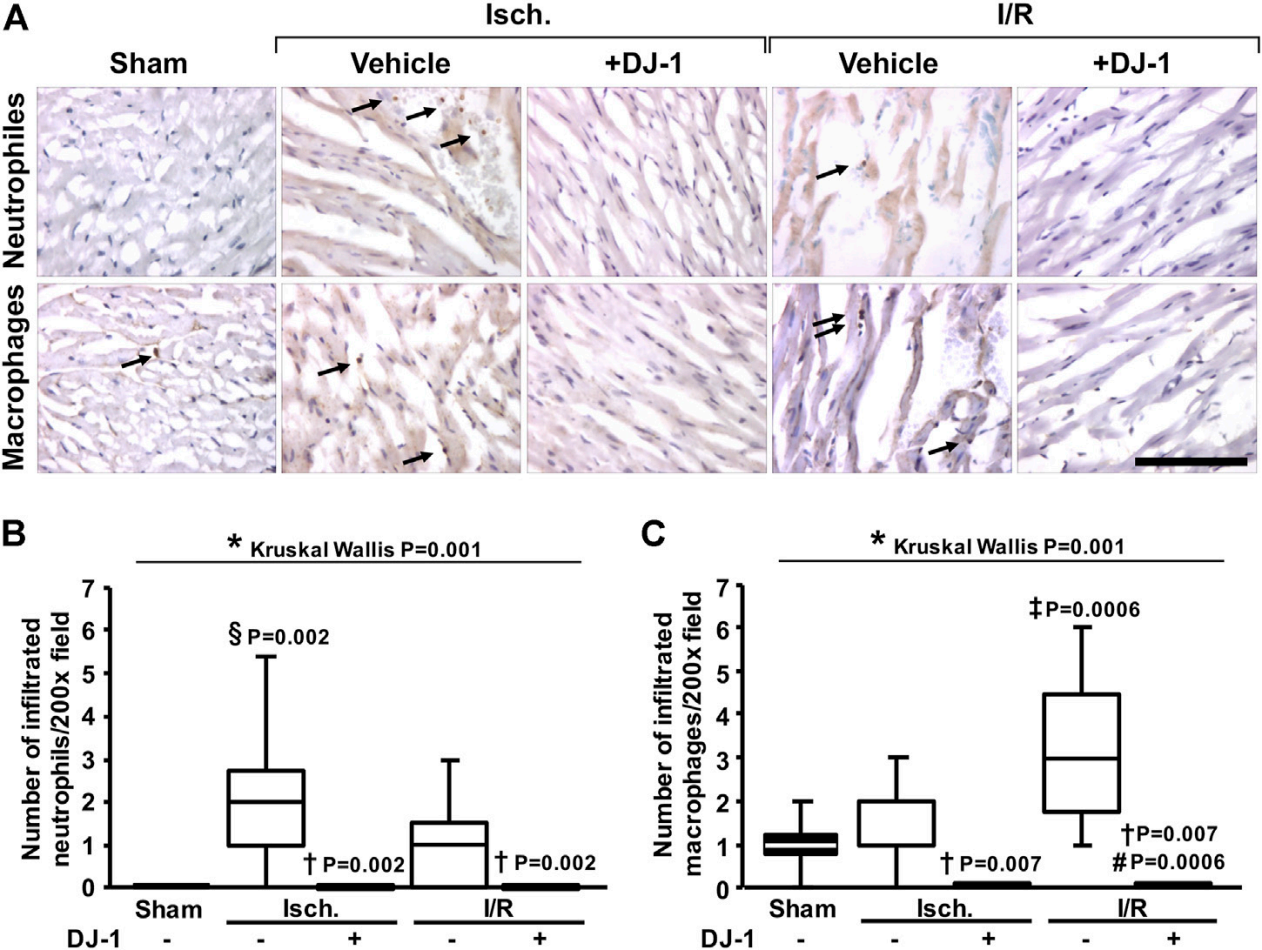
Effects of DJ-1 systemic administration on myocardial leukocyte infiltration after ischemia and ischemia/reperfusion. **(A)**. Representative immunohistochemical acquisitions of myocardial neutrophils (upper panel) and macrophages (lower panel) after ischemia and ischemia/reperfusion in the presence and the absence of a systemic administration of DJ-1. A sham-operated group was included as baseline. **(B)** and **(C)** quantification of infiltrated leukocytes. Scale bar: 100 µm; *p < 0.05, Kruskal-Wallis; §*p* < 0.05 vs. sham, †*p* < 0.05 vs. ischemia without DJ-1, ‡*p* < 0.05 vs. ischemia + DJ-1, #*p* < 0.05 vs. I/R without DJ-1, Dunn’s test. Isch., Ischemia; I/R, Ischemia/Reperfusion.

In order to functionally validate the predicted effects of DJ-1 administration in modulating the immune response to I/R, we quantified the leukocyte infiltration by immunohistochemistry (Figure 3). As a result, animals treated with DJ-1 exhibited significantly lower infiltration of neutrophils and macrophages, both after ischemia and I/R.

### Anti-apoptotic effects of DJ-1 administration

**FIGURE 4:**
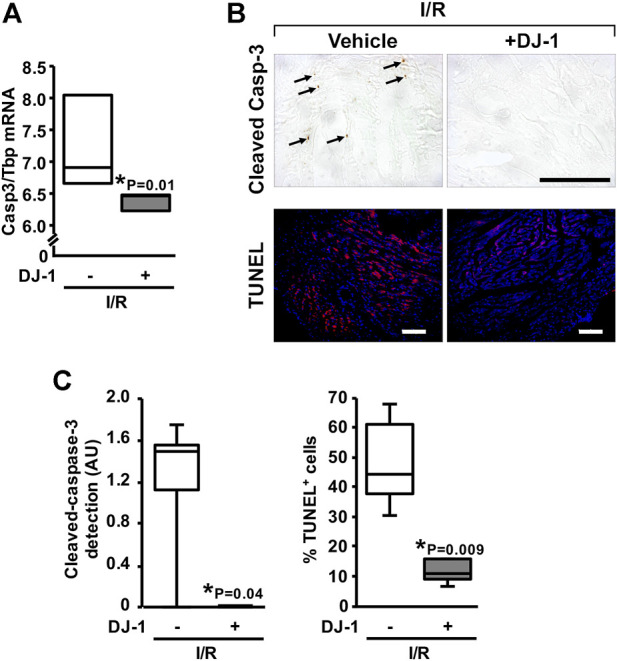
Effects of DJ-1 systemic administration on myocardial apoptosis after ischemia/reperfusion. **(A)** *Casp3* transcript analysis by qPCR. **(B)** Representative acquisitions of myocardial cleaved Casp-3 immunohistochemistry (upper panel) and TUNEL assay (lower panel) after ischemia/reperfusion. **(C)** myocardial cleaved Casp-3 immunohistochemistry and TUNEL positive cells quantification. Scale bar: 100 µm; *p < 0.05, Mann-Whitney. I/R, Ischemia/Reperfusion.

An effect of a systemic DJ-1 administration upon the induction of apoptosis following myocardial I/R was further investigated. Notably, animals treated with DJ-1 exhibited a diminished expression of *Casp3* gene after I/R, as assayed by qPCR (Figure 4A). Consistently, we could detect a significant reduction of the cleaved-Casp-3 myocardial content (*p* = 0.04), as well as the decrease of the TUNEL-positive cells (*p* = 0.009) following I/R for the DJ-1 treated group (Figure 4B,C).

### Anti-oxidant effects of DJ-1 administration

**FIGURE 5:**
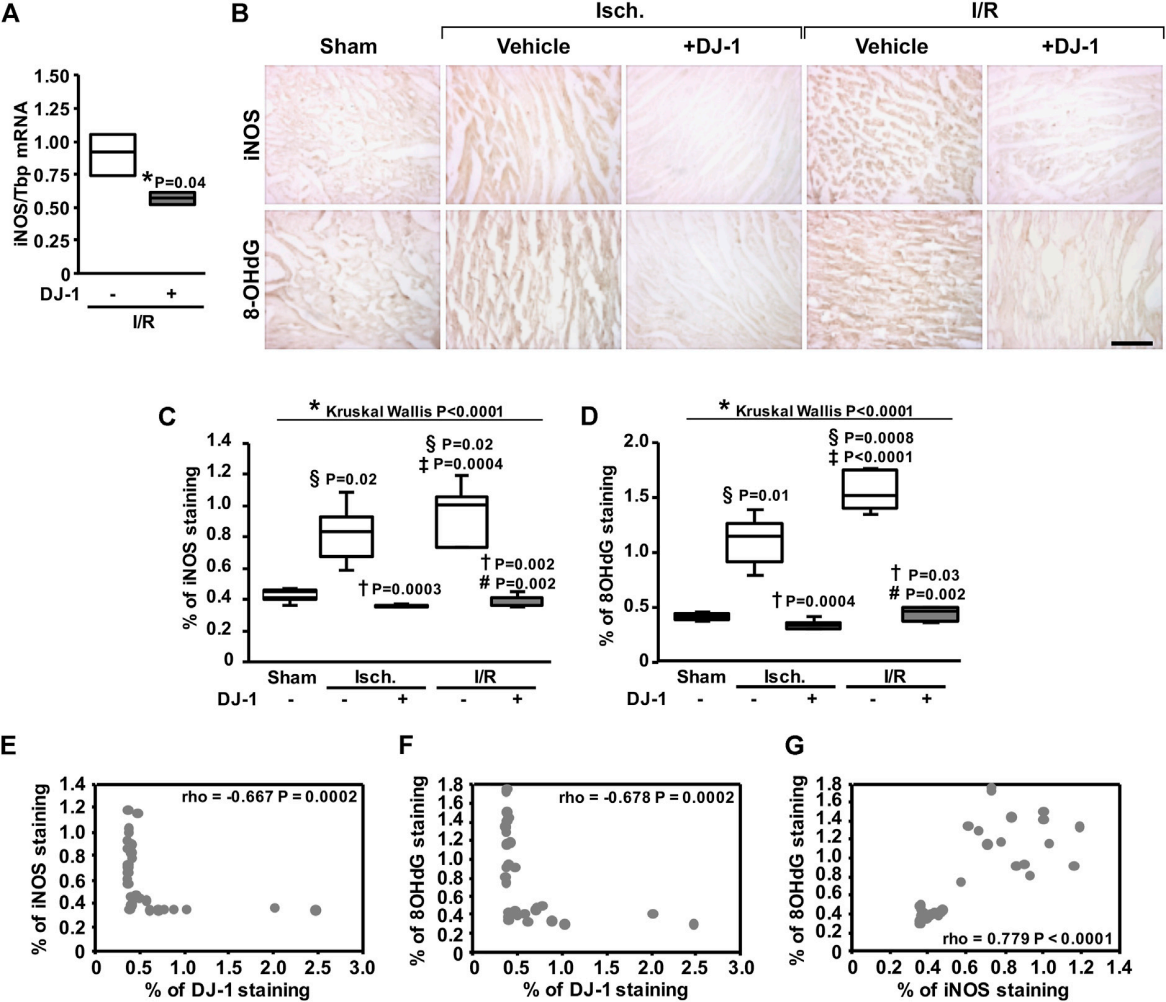
Systemic DJ-1 administration effects on oxidative stress after ischemia and ischemia/reperfusion. **(A)** *Inos* transcript analysis by qPCR. **(B)** Representative immunohistochemical acquisitions of myocardial iNOS (upper panel) and 8-OHdG (lower panel) after ischemia and ischemia/reperfusion in the presence and the absence of a systemic administration of DJ-1. A sham-operated group was included as baseline. **(C)** Myocardial iNOS content quantification. **(D)** Myocardial 8-OHdG content quantification. **(E–G)** Correlation analyses between iNOS and DJ-1 **(E)**, 8-OHdG and DJ-1 **(F)**, and iNOS and 8-OHdG **(G)** staining signals. Scale bar: 100 µm; *p < 0.05, Kruskal-Wallis; §*p* < 0.05 vs. sham, †*p* < 0.05 vs. ischemia without DJ-1, ‡*p* < 0.05 vs. ischemia + DJ-1, #*p* < 0.05 vs. I/R without DJ-1, Dunn’s test; rho, Spearman rank correlation coefficient. Isch., Ischemia; I/R, Ischemia/Reperfusion.

Because ROS-induced damage has a central role in I/R injury, we evaluated whether a systemic administration of DJ-1 has an effect upon the oxidative damage to the myocardium following I/R. As a result, the administration of DJ-1 significantly reduced the expression level of *Inos* gene, as well as the staining of the oxidative damage marker 8-hydroxy-deoxy-Guanine (8-OHdG) (Figure 5). Additionally, we found a negative correlation between the staining signals of DJ-1 and iNOS (Figure 5E), and between DJ-1 and 8-OHdG (Figure 5F). A positive correlation between iNOS and 8-OHdG staining was also evidenced (Figure 5G).

## Discussion

In the present study, we explored the effects of a systemic administration of recombinant DJ-1 upon ischemia and I/R injury in a mouse model of acute MI. We report a significant reduction in infarct size, leukocyte infiltration, apoptosis, and oxidative stress associated with the treatment. Also, we analysed the transcriptional response of the myocardium.

A role for the endogenous DJ-1 in cardioprotection has already been proven by gain- and loss-of-function studies. Hence, while DJ-1 deficiency do not affect cardiac performance at baseline, animals lacking DJ-1 exhibited larger infarcts, increased mitochondrial fission, and worse left ventricular function upon LAD ligation, compared to wild-type littermates (Dongworth et al., 2014; Kaneko et al., 2014; Shimizu et al., 2016; Dong et al., 2018; Xin et al., 2019). Similar results were found in experimental models of stroke, where the lack of DJ-1 resulted in larger infarcts *in vivo*, and enhanced cell death *in vitro* (Aleyasin et al., 2007)*.* In a previous study, we have described the up-regulation of DJ-1 following post-conditioning in a pre-clinical model of MI (Gallinat et al., 2022). In pressure overload animal models, mice lacking DJ-1 exhibited a higher oxidative stress level, exaggerated cardiac hypertrophy, and were more prone to develop heart failure (Billia et al., 2013). Collectively reinforcing a cardioprotective role for DJ-1.

Over the past decades, DJ-1 have been extensively studied, and many functions have been reported. These include chaperone (Shendelman et al., 2004), protease (Chen et al., 2010), and deglycase (Richarme et al., 2015; Richarme and Dairou, 2017) activities, regulation of transcription (Takahashi-Niki et al., 2017), redox sensing (Wilson, 2011) and the modulation of mitochondrial homeostasis (Hayashi et al., 2009; Heo et al., 2012). However, the exact molecular function of DJ-1, as well as its dynamics and regulation remain elusive. Interestingly, some reports suggest an extracellular activity, as it is secreted under some pathologic conditions such as breast cancer (Le Naour et al., 2001), Parkinson’s disease (Maita et al., 2008; Tsuboi et al., 2008) and stroke (Allard et al., 2005). Also, a pro-survival role for the extracellular form, has been reported in ischemia (Kaneko et al., 2014) and I/R (Han et al., 2017). Likewise, we previously described the endothelial secretion of DJ-1 during ischemia and I/R, and evidenced a role in regulating endothelial cell function (Gallinat and Badimon, 2022). The data here reported support a cardioprotective role for DJ-1 in MI, presumably for the extracellular form.

ROS are a group of small molecules derived from the reduction of the oxygen molecule, that are continuously produced in small amounts as by-products of cell respiration and metabolism, and eliminated by the endogenous antioxidant systems. Within the heart, ROS play a role as second messengers for the excitation-contraction coupling, cell differentiation, and regulation of blood flow (Forman et al., 2004; Burgoyne et al., 2012). However, the accumulation of ROS or the unbalance between ROS production and the antioxidant mechanisms (termed, oxidative stress) is detrimental and cause several macromolecular modifications, such as, lipid peroxidation, protein misfolding, and DNA damage. Indeed, oxidative stress is involved in the aetiology of a number of pathologies and cellular insults. At the onset of reperfusion, the reintroduction of O_2_ cause a burst of ROS within the mitochondria (Jassem et al., 2002), which challenges the antioxidant mechanisms and impairs the mitochondrial electron transport chain. This compromise the mitochondrial function, the ATP production and cell viability (Arduini et al., 1988). The significant decrease in the 8-OHdG staining signal following I/R for the treated animals, indicates that the administration of DJ-1 mitigates the oxidative damage to the myocardium. This observation was reinforced by the significant negative correlation found between DJ-1 and 8-OHdG staining signals.

In order to highlight possible mechanisms at play, we performed a whole-transcriptome analysis of the myocardium. Unexpectedly, we could not detect any of the previously described functions of DJ-1 enriched in the transcriptomic analysis, which suggests a distinct role for the extracellular and intracellular forms of DJ-1. GPCR-mediated signaling and immune response were rather the most consistently detected enrichments.

A body of evidences has linked GPCR signaling to cardioprotection. Endogenous GPCR ligands released during ischemia and reperfusion (i.e., autacoids) have been proposed as triggers of the cardioprotection conferred by ischemic pre- and post-conditioning (Heusch, 2015). This is the case for bradykinin (Goto et al., 1995; Oldenburg et al., 2004), opioids (Schultz et al., 1995; Zatta et al., 2008), acetylcholine (Yao and Gross, 1993), catecholamines (Tsuchida et al., 1994), angiotensin II (Liu et al., 1995), and endothelin-1 (Wang et al., 1996). Also, a number of GPCR agonists have proven to protect the heart against I/R injury.

GPCRs couple to a membrane-anchored heterotrimeric G_αβγ_ protein. Upon activation, GPCRs undergo a conformational change that allows the exchange of a GDP molecule, bound to the Gα subunit, for a GTP molecule, activating the G_α_ subunit. Thereafter, G_α_ dissociates from the remaining G_βγ_ subunits and triggers downstream signaling. GPCRs can be classified depending on the downstream effects of the activated G_α_ subunit into: G_αs_, which stimulate the adenylate cyclase, G_αi/o_, which inhibit adenylate cyclase, G_αq/11_, which activate phospholipase C, and G_α12/13_ which regulate Rho GTPase activity. Most of the GPCRs that play a role in cardioprotection couple to G_αi/o_ or G_αq/11_ proteins (Heusch, 2015). Furthermore, GPCR kinases, and β-arrestins, that are also involved in GPCR-mediated signaling (Gurevich and Gurevich, 2019), play a role in regulating cardiac injury upon MI (Brinks et al., 2010; Wang et al., 2017). The systemic administration of DJ-1 induced the up-regulation of several GPCRs following MI, some of them belonging to the odorant GPCRs family. Although still poorly understood, olfactory receptors have been reported to be expressed in the heart and to play a role in regulating cardiac function and angiogenesis (Kim et al., 2015; Jovancevic et al., 2017).

From the onset of ischemia to reperfusion and infarct healing, the immune response has a central role (Vilahur and Badimon, 2014; Ong et al., 2018). The delicate balance between pro-inflammatory and healing signals exerts a great impact on the outcome. As a matter of fact, several cardioprotective strategies have focused on the modulation of the immune response (Grilo et al., 2017; Andreadou et al., 2019; Zuurbier et al., 2019). Importantly, both pro- and anti-inflammatory signals are needed for the optimal resolution of MI (Yap et al., 2019; Sun et al., 2021). For the treated animals, we could detect several pathways related to the immune response enriched in the myocardium after MI. We further report, a diminished leukocyte infiltration following I/R for the treated animals. Amongst the immune response related pathways detected, the tumour necrosis factor α (TNFα) signaling pathway was the most enriched. Interestingly, TNFα signaling has been shown to display cardioprotection through NFκB-mediated cardiomyocyte expression of keratin-8 and keratin-18, which preserve the integrity and function of the intercalated discs and mitochondria upon stress (Papathanasiou et al., 2015).

Collectively our results illustrate a cardioprotective role for the exogenously administration of DJ-1 upon MI, potentially mediated by GPCRs signaling and the modulation of the immune response. The described extracellular activities of DJ-1 offer a therapeutic opportunity to limit I/R induced damage, which goes beyond MI. Whilst the present study provides the first line of evidence for an extracellular DJ-1 activity *in vivo*, the therapeutic potential of a systemic administration of recombinant DJ-1, requires further investigation regarding possible off-target activities of DJ-1. Also, long-term studies are needed to evaluate whether DJ-1 protective effects translate into the amelioration of adverse cardiac remodeling.

## Limitations

The present study provides the first line of evidence on the infarct limiting effects of administering recombinant DJ-1 on a mouse model of acute myocardial infarction. This study needs further investigation to advance our understanding of DJ-1 target and off-target effects as well as the tentative side effects of recombinant DJ-1 preparations. A major concern for the clinical use of recombinant proteins is their potential to induce an unwanted immune response that may compromise safety. Furthermore, while infarct size is widely recognized as a major predictor of clinical outcomes, long-term effects have not yet been addressed. Further investigation, in appropriated models is warranted in order to assess whether the cardioprotective effects of DJ-1 administration are maintained in the long-term.

## Data Availability

The datasets presented in this study can be found in online repositories. The names of the repository/repositories and accession number(s) can be found below: https://www.ncbi.nlm.nih.gov/geo/, GSE66307.
